# Association of circulating miR-20a, miR-27a, and miR-126 with non-alcoholic fatty liver disease in general population

**DOI:** 10.1038/s41598-019-55076-z

**Published:** 2019-12-11

**Authors:** Yoshitaka Ando, Mirai Yamazaki, Hiroya Yamada, Eiji Munetsuna, Ryosuke Fujii, Genki Mizuno, Naohiro Ichino, Keisuke Osakabe, Keiko Sugimoto, Hiroaki Ishikawa, Koji Ohashi, Ryoji Teradaira, Yoshiji Ohta, Nobuyuki Hamajima, Shuji Hashimoto, Koji Suzuki

**Affiliations:** 1 Department of Biomedical and Analytical Sciences, Fujita Health University School of Medical Sciences, 1-98, Dengakugakubo, Kutsukake-cho, Toyoake, Aichi 470-1192 Japan; 2 Department of Medical Technology, Kagawa Prefectural University of Health Sciences, 281-1, Murechohara, Takamatsu, Kagawa 761-0123 Japan; 3 Department of Hygiene, Fujita Health University School of Medicine, 1-98, Dengakugakubo, Kutsukake-cho, Toyoake, Aichi 470-1192 Japan; 4 Department of Biochemistry, Fujita Health University School of Medicine, 1-98, Dengakugakubo, Kutsukake-cho, Toyoake, Aichi 470-1192 Japan; 5 Department of Preventive Medical Sciences, Fujita Health University School of Medical Sciences, 1-98, Dengakugakubo, Kutsukake-cho, Toyoake, Aichi 470-1192 Japan; 6 Department of Joint Research Laboratory of Clinical Medicine, Fujita Health University Hospital, 1-98, Dengakugakubo, Kutsukake-cho, Toyoake, Aichi 470-1192 Japan; 7 Department of Clinical Physiology and Functional Imaging, Fujita Health University School of Medical Sciences, 1-98, Dengakugakubo, Kutsukake-cho, Toyoake, Aichi 470-1192 Japan; 8 Department of Chemistry, Fujita Health University School of Medicine, 1-98, Dengakugakubo, Kutsukake-cho, Toyoake, Aichi 470-1192 Japan; 9 Department of Healthcare Administration, Nagoya University Graduate School of Medicine, 65, Tsurumai-cho, Showa-ku, Nagoya, Aichi 466-8550 Japan

**Keywords:** Non-alcoholic fatty liver disease, Epidemiology, Biomarkers

## Abstract

Non-alcoholic fatty liver disease (NAFLD) is closely associated with obesity, metabolic syndrome, and type II diabetes mellitus. Recently, circulating microRNAs (miRNAs) have been proposed as useful disease biomarkers. We examined whether circulating miRNAs, such as miR-20a, miR-27a, and miR-126, were useful biomarkers for NAFLD. We conducted a cross-sectional analysis of 527 subjects aged 39 years or older who had undergone a health examination in the Yakumo Study. Of the residents, 92 were diagnosed with NAFLD using a registered medical sonographer. Serum miR-20a, miR-27a and miR-126 levels were measured by quantitative real-time PCR. We then calculated the odds ratios for serum miRNA level changes according to the severity of NAFLD using normal liver status as the reference group. Serum levels of miR-20a and 27a, but not miR-126, were significantly lower in NAFLD subjects than normal subjects. Serum miR-20a and miR-27a levels were significantly lower in both male and female severe NAFLD subjects. Logistic regression analysis showed a significant relationship between low circulating miR-20a and 27a levels and severe NAFLD. Down-regulated circulating miR-20a and 27a levels were significantly associated with severe NAFLD in the general population. Circulating miR-20a and miR-27a may be useful biomarkers for severe NAFLD.

## Introduction

Non-alcoholic fatty liver disease (NAFLD) currently represents one of the most common diseases worldwide. The global prevalence of NAFLD has increased from 20 to 30% over the past two decades. Although 80–90% of NAFLD patients have simple liver steatosis, the remaining 10–20% of patients have non-alcoholic steatohepatitis (NASH), which may progress to cirrhosis or even hepatocellular carcinoma^1^. Furthermore, NAFLD is closely associated with metabolic syndrome (MetS), type 2 diabetes mellitus (T2DM), and cardiovascular diseases (CVD)^2–4^.

Most subjects with NAFLD are often asymptomatic, and only detected incidentally when liver function tests or abdominal ultrasound are performed. Imaging method, such as ultrasonography (US), computed tomography (CT) and magnetic resonance imaging (MRI), is a noninvasive tool for early detection of NAFLD. However, these image methods are time-consuming and expensive, making the development of simpler methods necessary. Although liver biopsies are the gold standard for definitive diagnoses of hepatic steatosis, the potential risks of liver biopsies and the variability associated with sampling and interpretation make these procedures unsuitable for at-risk population screening. The discovery of novel screening methods remains challenging because no non-invasive disease biomarker can accurately and reliably distinguish between mild and severe disease stages. Thus, the development of a novel biomarker has been eagerly anticipated for a long time.

It is widely recognized that environmental and genetic factors interact to determine NAFLD phenotype and influence its progression^5^. Epigenetics can explain some of the relation between genes and external influences. The elucidation of epigenetic factors may facilitate the development of noninvasive biomarkers for the early detection while allowing for early preventive and therapeutic strategies for high-risk patients. The epigenetic changes that have been related to NAFLD are DNA methylation, histone modifications, and microRNA (miRNA) regulation^6–9^. MiRNAs are non-coding RNAs and small RNAs that consist of 20–25 nucleotides^10^. MiRNAs help regulate gene expression at post-transcription levels and are related to cell differentiation, apoptosis, and metabolism^10^. In addition, miRNAs are stable within bodily fluids (particularly plasma and serum) in which ribonuclease (RNase) molecules exist given that miRNAs are covered with small particles, including exosomes, high-density lipoproteins, and Argonaute, which protect miRNAs from degradation by RNase^11^. Recently, it has been reported that circulating miRNAs are associated with several diseases^7,12–16^. For example, we have reported that serum miR-21, miR-34a, miR-122 and miR-451 levels are elevated in middle-aged Japanese NAFLD patients and that the level of serum miR-122 is closely associated with the severity of liver steatosis in both male and female NAFLD patients^7^. Considering that the presence of more than 2,000 different miRNAs has been described in humans, it is perhaps not surprising that a comprehensive understanding of NAFLD in association with circulating miRNAs has not yet been achieved.

Dysregulated miRNA expression in the liver has been demonstrated in animal and human with NAFLD^17–19^. For example, mir-27a regulates hepatic lipid metabolism and alleviates NAFLD by decreasing the amount of fatty acid synthase and stearoyl-CoA desaturase 1. In addition, Feng *et al*. have reported that miR-126 may affect the pathogenesis of liver fibrosis^20^. MiR-20a regulates the proliferation and migration of hepatic cells^21^. Interestingly, these miRNAs can be detected from serum samples and have been proposed as attractive biomarkers. It is known that circulating miR-27 and miR-126 are associated with obesity, MetS and T2DM^22–27^. Additionally, we have reported that circulating miR-20a and miR-27a are negatively associated with obesity in the middle-aged general population. In addition, whereas NAFLD has been implicated in MetS-associated phenotypes, it is still unknown whether NAFLD patients present variations in their circulating miRNAs. Given that miRNAs can be detected from serum samples, miR-20a, miR-27a and miR-126 may be reflective of hepatic physiological conditions.

In the present study, we examined whether circulating miR-20a, miR-27a and miR-126 were associated with NAFLD and whether theses circulating miRNAs are useful NAFLD biomarkers.

## Results

Study subjects were excluded based on the following criteria: subjects who declined to participate in this research, missing dates for analysis, technical issues with sample vials, and a history of cancer or CVD. The characteristics of 475 subjects that were enrolled in the present study are shown in Table 1. Of 475 subjects, 92 subjects (male: n = 53, female: n = 39) were diagnosed with NAFLD. The body mass index (BMI), systolic blood pressure (SBP), diastolic blood pressure (DBP), Hemoglobin A1c (HbA1c), serum glucose, total protein, albumin, triglyceride (TG), low-density lipoprotein cholesterol (LDL-c), AST, ALT, and γ-glutamyl transpeptidase (γGT) levels were significantly higher in NAFLD subjects than in normal subjects. Serum high-density lipoprotein cholesterol (HDL-c) levels were significantly lower in NAFLD subjects than in normal subjects. The number of smokers among NAFLD subjects was significantly higher than among normal subjects (*p* < 0.05).

**Table 1:** Characteristics of study subjects.

	Normal	NAFLD	*P* value
Male/Female^a^	129/254	53/39	<0.01
Age (years)^b^	64.1 ± 10.3	62.9 ± 8.9	0.33
BMI (kg/m^2^)^b^	22.85 ± 3.02	26.1 ± 2.84	<0.01
SBP (mmHg)^b^	132.6 ± 19.1	140.4 ± 15.9	<0.01
DBP (mmHg)^b^	74.4 ± 11.3	81.3 ± 12.0	<0.01
HbA1c (%)^b^	5.41 ± 0.47	5.78 ± 0.98	<0.01
Glucose (mg/dl)^b^	90.4 ± 14.8	98.4 ± 26.1	<0.01
Total protein (g/dl)^b^	7.44 ± 0.43	7.63 ± 0.37	<0.01
Albumin (g/dl)^b^	4.43 ± 0.27	4.55 ± 0.24	<0.01
TG (mg/dl)^c^	87 (62–114)	127 (89–184)	<0.01
Total cholesterol (mg/dl)^b^	211.1 ± 31.2	214.8 ± 34.8	0.32
HDL-c (mg/dl)^b^	61.1 ± 13.7	52.5 ± 10.1	<0.01
LDL-c (mg/dl)^b^	122.8 ± 28.5	130.1 ± 32.9	0.03
ALP (IU/l)^c^	216 (181–258)	220 (190–231)	0.47
AST (IU/l)^c^	22.2 (19–25)	25.5 (21.0–30.8)	<0.01
ALT (IU/l)^c^	19.7 (15–25)	29.1 (20.0–39.0)	<0.01
γGT (IU/l)^c^	23.9 (15.0–33.0)	35.0 (22.3–55.5)	<0.01
eGFR(ml/min/1.73 m^2^)^b^	67.48 ± 13.83	73.34 ± 12.79	<0.01

^a^Date are compared by chi-square test.

^b^Date are expressed as mean value standard deviation and compared by student t-tests.

^c^Date are expressed as geometric mean value (25th-75th percentiles) and compared by Wilcoxon tests.

**Figure 1:**
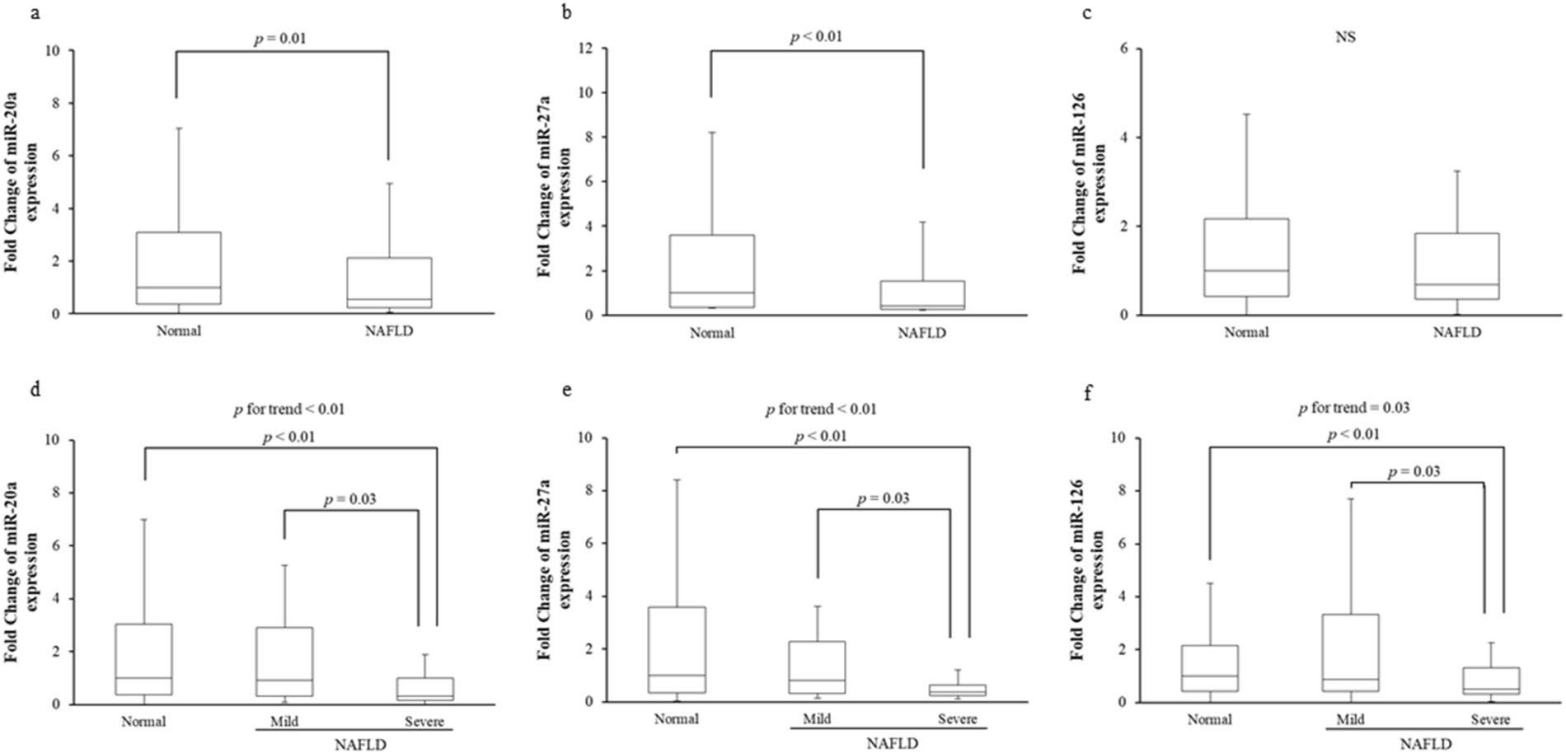
Quantitative real-time PCR analysis of circulating levels for three miRNAs (miR-20a, miR-27a and miR-126) in two groups (normal and NAFLD) (**a–c**) or three groups (normal, mild and severe NAFLD) (**d–f**). NS, not statistically significant.

We examined whether the levels of circulating miR-20a, miR-27a, and miR-126, which are known to be associated with obesity, MetS, and/or T2DM^14,22–28^, were altered in NAFLD subjects. Serum miR-20a and miR-27a levels were significantly lower in NAFLD subjects than in normal subjects (Fig. 1a,b). There was no significant difference in serum miR-126 levels between NAFLD and normal subjects, although serum miR-126 trended to be lower in NAFLD subjects than in normal subjects (Fig. 1c). Next, we examined whether low circulating miR-20a, miR-27a, and miR-126 were associated with disease severity in NAFLD subjects. Serum miR-20a, miR-27a, and miR-126 levels were significantly lower in subjects with severe NAFLD compared to normal or mild NAFLD subjects (Fig. 1d–f).

**Figure 2:**
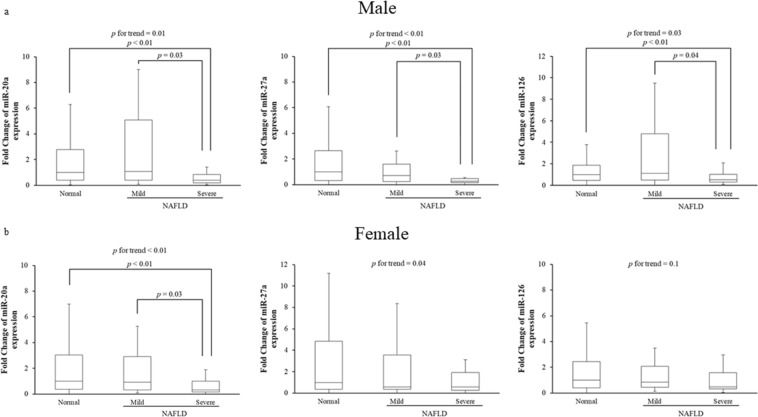
Quantitative real-time PCR analysis of circulating levels for three miRNAs (miR-20a, miR-27a and miR-126) in NAFLD patients (**a:** male, **b**: female). Subjects with NAFLD were divided into two groups (mild and severe). NS, not statistically significant.

We further examined whether the association between low serum miR-20a, miR-27a, and miR-126 levels and NAFLD disease severity was affected by the sex of the subject. Serum miR-20a levels were significantly lower in both male and female subjects with severe NAFLD compared to normal subjects (Fig. 2a,b). For male subjects, serum miR-27a levels were significantly lower in severe NAFLD subjects than in normal or mild NAFLD subjects, and serum miR-126 levels were significantly lower in severe NAFLD subjects compared to normal or mild NAFLD subjects (Fig. 2a). For female subjects, there were no significant differences in serum miR-27a levels between severe NAFLD subjects or either normal or mild NAFLD subjects, while significant decreasing trend is observed (*p* for trend = 0.04), but there were no significant differences in serum miR-126 levels among the three groups (*p* for trend = 0.1) (Fig. 2b).

We assessed the severity of liver disorder by a method different from US. Fibrosis-4 index (FIB-4) is hepatic fibrosis biomarker. We confirmed the correlation between circulating miRNAs levels and FIB-4 score. The circulating miR-20a and 27a levels were not correlated with FIIB-4 score. However, the inverse correlation (although slight) was found between FIB-4 score and circulating miR-126 levels (Supplementary Table S1). In addition, we confirmed whether circulating miRNAs levels are associated with advancing liver fibrosis when we divided all subjects into three groups by FIB-4 score. There was no significant difference in each serum miRNA level among the three groups (Supplementary Table S2).

The relationships between low serum miR-20a, miR-27a, and miR-126 levels and disease severity in all NAFLD subjects were further examined by logistic regression analysis. Table 2 shows that the association between low serum miRNAs levels and NAFLD severity was adjusted for age and sex. The odds ratios (ORs) low levels of miR-20a, miR-27a, and miR-126 in serum were significantly higher in severe NAFLD subjects than in normal subjects. Next, to exclude multiple factors associated with NAFLD, we performed a logistic regression analysis that yielded a model excluding age, sex, BMI*,* SBP, HbA1c, TG, LDL-c, the estimated glomerular filtration rate (eGFR), smoking history and medications. The results of this model are shown in Table 3. For all NAFLD subjects, the ORs between mild NAFLD and low serum miR-20a, miR-27a, and miR-126 levels were not statistically significant. However, the ORs between severe NAFLD subjects and low serum miR-20a and miR-27a levels were statistically significant, whereas the odds ratio between severe NAFLD and low serum miR-126 levels was not.

**Table 2:** Age- and sex- adjusted ORs and 95% CIs for down-regulated circulating miRNAs according to Normal status.

miRNAs	Normal n = 383	Mild NAFLD n = 41	*P* value	Severe NAFLD n = 51	*P* value
miR-20a	1.0	0.89 (0.39–1.88)	0.78	4.34 (2.27–8.37)	<0.01
miR-27a	1.0	1.00 (0.45–2.06)	0.99	3.29 (1.73–6.29)	<0.01
miR-126	1.0	0.63 (0.25–1.39)	0.27	3.29 (1.73–6.30)	0.01

**Table 3:** Multivariate adjusted ORs and 95% CIs for down-regulated circulating miRNAs according to Normal status.

miRNAs	Normal n = 383	Mild NAFLD n = 41	*P* value	Severe NAFLD n = 51	*P* value
miR-20a	1.0	1.14 (0.45–2.70)	0.78	4.09 (1.70–10.14)	<0.01
miR-27a	1.0	1.28 (0.52–2.98)	0.58	2.53 (1.04–6.10)	0.04
miR-126	1.0	0.93 (0.34–2.31)	0.88	1.63 (0.66–3.91)	0.28

ORs and 95% CIs were adjusted for age, sex, BMI, SBP, HbA1c, LDL-c, TG, eGFR, smoking history and medications.

## Discussion

In this study, we found that low levels of the three circulating miRNAs (miR-20a, miR-27a, and miR-126) were related to severe NAFLD for all subjects. Specifically, lower circulating miR-20a and miR-27a levels were found in severe NAFLD subjects. Down-regulated circulating miR-20a and miR-27a were associated with severe disease in NAFLD subjects, whereas down-regulated circulating miR-126 was associated with severe disease in male NAFLD subjects.

It is well known that levels of circulating miRNAs in humans vary widely according to several factors such as age, gender, or race^29–31^. Given that circulating miRNAs are affected by several elements, the existence of confounding factors should be considered. That is to say, we need to investigate the levels of circulating miRNAs in the general population. Our logistic regression results show an association between down-regulated circulating miR-20a, miR-27a, and miR-126 levels and disease severity in NAFLD subjects. Although we adjusted for lifestyle risk factors, such as smoking and exercise, circulating miR-20a and miR-27a were significantly associated with NAFLD (Tables 2 and 3). These results suggest that circulating miR-20a and miR-27a levels may represent novel factors for NAFLD that are independent of well-known lifestyle-related factors.

Over the past few years, several studies have been carried out that have focused on circulating miRNAs in NAFLD patients. Specifically, Liu *et al*. reported that some circulating miRNAs, such as miR-122, are approximately 2-fold higher in NAFLD samples. In addition, Tan *et al*. have showed that the levels of serum miRNAs, such as miR-122, are altered in NAFLD patients^32^. In contrast to previous results, the current study considered the general population. To date, no study has attempted to investigate the relationships between circulating miRNAs and NAFLD in general population except for our research. It should be noted that samples from the general population were able to provide clarity regarding lower circulating miRNA levels, indicating that future studies that consider the general population may be more successful when screening for NAFLD biomarkers.

To date, several studies have demonstrated that NAFLD may change circulating miRNA expression^14,15,33,34^. For example, NAFLD patients show the up-regulated expression of circulating miRNAs, such as miR-21, miR-34a, miR-122, and miR-451^7^. Another study has also shown that NAFLD increases with circulating miR-34a and miR-192 expression^35^. Additionally, Salvoza N *et al*. showed up-regulated miR-34a and miR-122 expression in NAFLD patients^36^. It should be noted that the current study found lower levels of miRNAs, such as miR-20a, miR-27a, and miR-126. Generally, circulating miRNA expression is normalized by U6 levels or spiked-in cyn-39 levels due to the lack of an established internal standard for circulating miRNA. Thus, results regarding variations in circulating miRNA levels may be affected depending on the selection of internal standards. As such, the ratio of down-regulated to up-regulated miRNA may be more useful in distinguishing between normal and NAFLD patients in the absence of an internal standard. Indeed, Deng Y *et al*.reported that the ratio-based method can solve the normalization problem for circulating miRNAs^37^.

FIB-4 is one of biomarkers for liver fibrosis^38^. In order to elucidate whether the levels of circulating miR-20a, 27a and 126 are not associated with liver fibrosis, we examined the association of these circulating miRNAs levels and FIB-4 score. As a result, there was a weak inverse correlation between circulating miR-126 level and FIB-4 score. There is a report showing that hepatic miR-126 level is decreased in patients with NASH^39^. On the other hand, circulating miR-20a and 27a levels were not correlated with neither FIB-4 score nor advancing liver fibrosis. To the best of our knowledge, there were no reports showing that decreased circulating miR-20a and 27a levels are associated with liver fibrosis. Therefore, we suggested that decreased circulating miR-20a and 27a levels could reflect intrahepatic steatosis, but not liver fibrosis.

In the present study, the levels of serum miR-20a and miR-27a, which are associated with obesity, MetS, and T2DM^14,22–24^, were found to be significantly lower in NAFLD subjects. It has been reported that NAFLD shows ectopic fat accumulation that is usually accompanied by decreased glycogen synthesis^40^. Hepatic glycogen synthesis is stimulated by miR-20a^41^. Down-regulated circulating miR-20a levels were associated with reduced hepatic glycogen synthesis. However, Dan *et al*. reported that circulating miR-20a was up-regulated in T2DM patients with NAFLD complication^42^. The reason for the difference between their report and our results is unclear at present. In the present study, NAFLD subjects had not been diagnosed with T2DM, although the NAFLD subjects had significantly higher (although slight) fasting serum glucose and HbA1c levels than normal subjects. Therefore, the differences regarding circulating miR-20a levels in NAFLD patients between their report and our study may be due to differences in T2DM severity.

It has been demonstrated that miR-27a is related to lipid metabolism in the liver^43^. Zhang M *et al*. reported that hepatic miR-27a plays a critical role in lipid homeostasis of the liver and that the overexpression of miR-27a attenuated NAFLD development^44^. In addition, it has been reported that the expression of miR-27a decreases PPARγ expression^45^. PPARγ regulates lipogenic and adipogenic gene expression and is related to the pathophysiology of obesity. In this study, we indicated that low levels of circulating miR-27a were associated with severe NAFLD. Given the results of previous research, we suggest that the decreased expression of circulating miR-27a may result in increased PPARγ expression in the liver.

In the present study, we found that the low levels of three circulating miRNAs (i.e., miR-20a, miR-27a, and miR-126) were associated with NAFLD. Nevertheless, there are some limitations to our study. NAFLD in the recruited subjects was diagnosed by US, because we recruited subjects from health examination participants and because it is known that US is a reliable and accurate method to be able to detect moderate-severe fatty liver as much as histological examination^46^. Liver biopsy is the gold standard for NAFLD diagnosis. Therefore, further studies are required to establish the association of the levels of serum miRNAs with the severity of NAFLD diagnosed by liver biopsy. Published studies, including our present study, lack long-term outcome data regarding disease activity, prognostic expectations, and pathogenesis. In addition, we recruited only Japanese subjects in this study, which may have potentially biased our results towards a particular ethnic group. The inclusion of other ethnic groups in future studies may result in different circulating miRNA profiles. Therefore, further studies are required to elucidate whether down-regulated circulating miR-20a and miR-27a may be used as novel biomarkers for severe NAFLD and whether a combination of circulating miR-20a and/or miR-27a with other circulating miRNAs enhances the sensitivity of the disease severity-dependent detection of NAFLD.

In conclusion, the results of the present study indicate that down-regulated circulating miR-20a, miR-27a, and miR-126 are associated with severe NAFLD in the Japanese general population, although the association of down-regulated circulating miR-20a and miR-27a levels with severe NAFLD seem to be superior to that of down-regulated circulating miR-126 levels. We suggest that down-regulated circulating miR-20a and miR-27a may be useful biomarkers for severe NAFLD.

## Materials and Methods

### Study subjects

A community-based health examination is conducted annually at the end of August in the town of Yakumo, Hokkaido, in northern Japan. Volunteers that reside in Yakumo and that are aged 39 years or older during the health-examination were eligible to participate in our study. Residents who chose to not participate in the study or those who could not complete the lifestyle questionnaire were excluded as research subjects. This cross-sectional study is part of the larger Yakumo study, which is a population-based prospective study conducted in the area. In this study, we recruited 527 residents who participated in the 2012 Yakumo study. All participants provided written informed consent. The study protocol was approved by the Ethics Committee of Fujita Health University (approval number 164) and complied with guidelines of the Declaration of Helsinki.

### Participant data

A trained public health nurse gathered background information for each participant that included smoking habits (i.e., never, former, or current), alcohol consumption, and a history of major illnesses (i.e., yes/no) using self-administered questionnaires during the health examination. In addition, height, weight, waist circumference, and blood pressure were measured during the health examination. The BMI was calculated by dividing weight (kg) by height squared (m^2^). Participants with alcohol consumption ≥20 g/day (females) or ≥30 g/day (males) were excluded from the study, as the recommendations for NAFLD restrict alcohol consumption^19^.

### Assessment of hepatic steatosis

The presence of intrahepatic steatosis was assessed by US using a ProSound α7 with a UST-9130 convex probe (Hitachi Aloka Medical, Ltd. Tokyo, Japan) operated by three registered medical sonographers (Japan Society of Ultrasonics in Medicine). The ultrasonography images were independently reviewed by each sonographer who graded the degree of hepatic steatosis as either normal, mild, or severe^47^. Mild steatosis was defined as a slight increase in liver echogenicity and hepato-renal echo contrast. Severe steatosis was defined as a definite or marked increase in hepatic echogenicity and a poor or lack of visualization of the hepatic vessels and diaphragm. When assessments between observers differed, an agreement of two out of three observers was required for diagnosis. Subjects with NAFLD were divided into two groups based on the degree of liver steatosis: mild (male: n = 22, female: n = 19) or severe (male: n = 31, female: n = 20).

### Blood biochemistry

Fasting blood samples were taken during the health examination and sera were separated from blood samples by centrifugation within 1 h of collection. Serum samples were stored in a deep freezer at −80 °C until analysis.

Glucose, total protein, albumin, TG, total cholesterol, HDL-c, LDL-c, AST, ALT, γGT in serum, and HbA1c in whole blood were assayed using an autoanalyzer in the laboratory of Yakumo General Hospital on the day of the health examination. According to our previous study^48^ eGFR was calculated from serum creatinine using the following equation: eGFR = 194 × age^−0.287^ × serum creatinine^−1.019^(×0.739 for female). FIB-4 is an easily available score to assess liver fibrosis^38^. This score was calculated by the following equation: FIB-4 = (Age [years] × AST(IU/L))/(platelet count(10^9^/L) × (ALT (IU/L))^1/2^). FIB-4 cutoff points are three points to discriminate on the severity of liver fibrosis. FIB-4 score under 1.3 points implies no presence of fibrosis. FIB-4 score upper 2.67 points implies the presence of liver fibrosis. FIB-4 score between 1.3 and 2.67 points implies a possible presence of fibrosis^49^.

We used quantitative real-time polymerase chain reaction (qPCR) to detect levels of expression for 3 miRNAs in the sera as previously described^7,50^. In brief, we used TRIzol reagent (Invitrogen, Carlsbad, CA, USA) to isolate serum miRNAs. TRIzol regent and serum were mixed and further mixed with 5 µl of 5 nM Syn-cel-miR-39 miRScript miRNA Mimic to provide normalized control; each sample was vortexed immediately. Each sample was added to molecular grade chloroform (FUJIFILM Wako Pure Chemical Corporation, Osaka, Japan) to separate the aqueous and organic phases; followed by centrifuging at 12,000 rpm for 15 min at 4 °C. The aqueous phase was immediately transferred to a new 1.5 mL tube, and the TRIzol protocol continued. Isolated total RNA was dissolved in 15 μl of RNase-free water. The reverse transcription reaction system contained 1 μl of miScript Reverse Transcriptase Mix, 4 μl of 5× miScript HiFlex Buffer, 5 μl of total RNA, and 10 μl of RNase-free water. Samples were allowed to react at 37 °C for 60 min followed by 95 °C for 5 min in a 2720 Thermal Cycler (Applied Biosystems, Foster City CA, USA). The reacted sample was diluted with 380 μl of TE Buffer (10 mM Tris-HCl, pH 8.0; 1 mM EDTA, pH 8.0). Quantitative real-time polymerase chain reaction (qRT-PCR) was performed with a miScript System (Qiagen, Valencia, CA, USA) that included specific primers for each miRNA. PCR cycles were completed at 95 °C for 15 min, 45 cycles at 94 °C for 15 s, 55 °C for 30 s, and 70 °C for 30 s. Real-time PCR was performed using an ABI PRISM 7900 Sequence Detection System (Applied Biosystems, Foster City, CA, USA). The relative expression of each miRNA was calculated using the comparative cycle threshold method (2^−ΔΔCT^) normalized to spiked-in cyn-39 levels^7,14,50^.

### Statistical analysis

Statistics were calculated using JMP ver. 10.0 software (SAS Institute, Cary, NC, USA). Analysis of variance (ANOVA) and the Wilcoxon tests were used to compare continuous parameters, whereas the 3 was used to compare categorical variables. Serum concentrations of TG, ALP AST, ALT, and γ-GT were represented as geometric means and 25th–75th percentile ranges. Other variables were represented as the means ± SD. Spearman’s rank correlation was used to assess the correlation of FIB-4 score with each circulating miRNA level.

Group serum miRNA levels were compared by Wilcoxon tests and Jonckheere-Terpstra test. We then performed multivariate logistic regression analyses to estimate adjusted odds ratios ORs with 95% confidence intervals (CIs) for each confounding factor. We calculated the ORs for elevated serum miRNA levels (less than the 25th percentile) by NAFLD status using the normal subjects as the reference group. Covariates for adjustment included age, sex, BMI, SBP, HbA1c, TG, LDL-c, eGFR, cigarette smoking status (i.e., never, former, and current) and medication history (e.g., anti-hypertension, anti-cardiac diseases, anti-diabetes mellitus, anti-liver diseases or anti-dyslipidemia). For all covariates, missing values were included in the model as an additional category variable. All statistical tests were 2-tailed, and p-values less than 0.05 were considered statistically significant.

## Supplementary information

Supplementary information

## References

[1] Hashimoto E, Taniai M, Tokushige K (2013). Characteristics and diagnosis of NAFLD/NASH. J Gastroenterol Hepatol.

[2] Polyzos SA, Kountouras J, Mantzoros CS (2019). Obesity and nonalcoholic fatty liver disease: From pathophysiology to therapeutics. Metabolism.

[3] Asrih M, Jornayvaz FR (2015). Metabolic syndrome and nonalcoholic fatty liver disease: Is insulin resistance the link?. Mol Cell Endocrinol.

[4] Mavrogiannaki AN, Migdalis IN (2013). Nonalcoholic Fatty liver disease, diabetes mellitus and cardiovascular disease: newer data. Int J Endocrinol.

[5] Eslam M, Valenti L, Romeo S (2018). Genetics and epigenetics of NAFLD and NASH: Clinical impact. J Hepatol.

[6] Yamazaki M (2016). Fructose consumption induces hypomethylation of hepatic mitochondrial DNA in rats. Life Sci.

[7] Yamada H (2013). Associations between circulating microRNAs (miR-21, miR-34a, miR-122 and miR-451) and non-alcoholic fatty liver. Clin Chim Acta.

[8] Ohashi K (2015). High fructose consumption induces DNA methylation at PPARalpha and CPT1A promoter regions in the rat liver. Biochem Biophys Res Commun.

[9] Del Campo José, Gallego-Durán Rocío, Gallego Paloma, Grande Lourdes (2018). Genetic and Epigenetic Regulation in Nonalcoholic Fatty Liver Disease (NAFLD). International Journal of Molecular Sciences.

[10] Bartel DP (2004). MicroRNAs: genomics, biogenesis, mechanism, and function. Cell.

[11] Cortez MA (2011). MicroRNAs in body fluids–the mix of hormones and biomarkers. Nat Rev Clin Oncol.

[12] Ma E, Fu Y, Garvey WT (2018). Relationship of Circulating miRNAs with Insulin Sensitivity and Associated Metabolic Risk Factors in Humans. Metab Syndr Relat Disord.

[13] Viereck J, Thum T (2017). Circulating Noncoding RNAs as Biomarkers of Cardiovascular Disease and Injury. Circ Res.

[14] Munetsuna E (2018). Association of subcutaneous and visceral fat with circulating microRNAs in a middle-aged Japanese population. Ann Clin Biochem.

[15] Pant K, Venugopal SK (2017). Circulating microRNAs: Possible role as non-invasive diagnostic biomarkers in liver disease. Clin Res Hepatol Gastroenterol.

[16] Enache LS (2014). Circulating RNA molecules as biomarkers in liver disease. Int J Mol Sci.

[17] Nie J, Li CP, Li JH, Chen X, Zhong X (2018). Analysis of nonalcoholic fatty liver disease microRNA expression spectra in rat liver tissues. Mol Med Rep.

[18] Wang L (2016). Decreased MiR-155 Level in the Peripheral Blood of Non-Alcoholic Fatty Liver Disease Patients may Serve as a Biomarker and may Influence LXR Activity. Cell Physiol Biochem.

[19] Yamada H (2015). Longitudinal study of circulating miR-122 in a rat model of non-alcoholic fatty liver disease. Clin Chim Acta.

[20] Feng X (2015). Upregulation of microRNA-126 in hepatic stellate cells may affect pathogenesis of liver fibrosis through the NF-kappaB pathway. DNA Cell Biol.

[21] Chen GS (2016). Restoration of miR-20a expression suppresses cell proliferation, migration, and invasion in HepG2 cells. Onco Targets Ther.

[22] Nunez Lopez YO, Garufi G, Seyhan AA (2016). Altered levels of circulating cytokines and microRNAs in lean and obese individuals with prediabetes and type 2 diabetes. Mol Biosyst.

[23] Villard, A., Marchand, L., Thivolet, C. & Rome, S. Diagnostic Value of Cell-free Circulating MicroRNAs for Obesity and Type 2 Diabetes: A Meta-analysis. *J Mol Biomark Diagn* **6** (2015). 10.4172/2155-9929.1000251 PMC4905583 27308097

[24] Karolina DS (2012). Circulating miRNA profiles in patients with metabolic syndrome. J Clin Endocrinol Metab.

[25] Zhang T (2015). Circulating miR-126 is a potential biomarker to predict the onset of type 2 diabetes mellitus in susceptible individuals. Biochem Biophys Res Commun.

[26] Liu Y (2014). The role of circulating microRNA-126 (miR-126): a novel biomarker for screening prediabetes and newly diagnosed type 2 diabetes mellitus. Int J Mol Sci.

[27] Zhang T (2013). Plasma miR-126 is a potential biomarker for early prediction of type 2 diabetes mellitus in susceptible individuals. Biomed Res Int.

[28] Hijmans JG (2018). Influence of Overweight and Obesity on Circulating Inflammation-Related microRNA. Microrna.

[29] Wang YT, Tsai PC, Liao YC, Hsu CY, Juo SH (2013). Circulating microRNAs have a sex-specific association with metabolic syndrome. J Biomed Sci.

[30] Olivieri F (2012). Age-related differences in the expression of circulating microRNAs: miR-21 as a new circulating marker of inflammaging. Mech Ageing Dev.

[31] Zhao H (2010). A pilot study of circulating miRNAs as potential biomarkers of early stage breast cancer. PLoS One.

[32] Tan Y, Ge G, Pan T, Wen D, Gan J (2014). A pilot study of serum microRNAs panel as potential biomarkers for diagnosis of nonalcoholic fatty liver disease. PLoS One.

[33] Hiratsuka I, Yamada H, Munetsuna E, Hashimoto S, Itoh M (2016). Circulating MicroRNAs in Graves’ Disease in Relation to Clinical Activity. Thyroid.

[34] Yamada H, Itoh M, Hiratsuka I, Hashimoto S (2014). Circulating microRNAs in autoimmune thyroid diseases. Clin Endocrinol (Oxf).

[35] Liu CH (2018). miRNAs in patients with non-alcoholic fatty liver disease: A systematic review and meta-analysis. J Hepatol.

[36] Salvoza NC, Klinzing DC, Gopez-Cervantes J, Baclig MO (2016). Association of Circulating Serum miR-34a and miR-122 with Dyslipidemia among Patients with Non-Alcoholic Fatty Liver Disease. PLoS One.

[37] Deng Y (2019). Ratio-Based Method To Identify True Biomarkers by Normalizing Circulating ncRNA Sequencing and Quantitative PCR Data. Anal Chem.

[38] Sterling RK (2006). Development of a simple noninvasive index to predict significant fibrosis in patients with HIV/HCV coinfection. Hepatology.

[39] Cheung O (2008). Nonalcoholic steatohepatitis is associated with altered hepatic MicroRNA expression. Hepatology.

[40] Byrne CD, Targher G (2015). NAFLD: a multisystem disease. J Hepatol.

[41] Fang W (2016). MicroRNA-20a-5p contributes to hepatic glycogen synthesis through targeting p63 to regulate p53 and PTEN expression. J Cell Mol Med.

[42] Ye D (2018). Plasma miR-17, miR-20a, miR-20b and miR-122 as potential biomarkers for diagnosis of NAFLD in type 2 diabetes mellitus patients. Life Sci.

[43] Ji J (2009). Over-expressed microRNA-27a and 27b influence fat accumulation and cell proliferation during rat hepatic stellate cell activation. FEBS Lett.

[44] Zhang M, Sun W, Zhou M, Tang Y (2017). MicroRNA-27a regulates hepatic lipid metabolism and alleviates NAFLD via repressing FAS and SCD1. Sci Rep.

[45] Lin Q, Gao Z, Alarcon RM, Ye J, Yun Z (2009). A role of miR-27 in the regulation of adipogenesis. FEBS J.

[46] Hernaez Ruben, Lazo Mariana, Bonekamp Susanne, Kamel Ihab, Brancati Frederick L., Guallar Eliseo, Clark Jeanne M. (2011). Diagnostic accuracy and reliability of ultrasonography for the detection of fatty liver: A meta-analysis. Hepatology.

[47] Strauss S, Gavish E, Gottlieb P, Katsnelson L (2007). Interobserver and intraobserver variability in the sonographic assessment of fatty liver. AJR Am J Roentgenol.

[48] Fujii, R. *et al*. Associations of circulating microRNAs (miR-17, miR-21, and miR-150) and chronic kidney disease in a Japanese population. *J Epidemiol*, 10.2188/jea.JE20180233 (2019). 10.2188/jea.JE20180233 PMC7064557 30905898

[49] Shah AG (2009). Comparison of noninvasive markers of fibrosis in patients with nonalcoholic fatty liver disease. Clin Gastroenterol Hepatol.

[50] Kondo M (2019). Associations of serum microRNA-20a, -27a, and -103a with cognitive function in a Japanese population: The Yakumo study. Arch Gerontol Geriatr.

