# Supplementary material for: Association of circulating miR-20a, miR-27a, and miR-126 with non-alcoholic fatty liver disease in general population
Source: Sci Rep. 2019 Dec 11;9:18856. doi: 10.1038/s41598-019-55076-z (PMC6906495; doi:10.1038/s41598-019-55076-z)
Supplement: Supplementary file 1 — Supplementary information [file 41598_2019_55076_MOESM1_ESM.docx]

Supplementary information

**Association of circulating miR-20a, miR-27a, and miR-126 with non-alcoholic fatty liver disease in general population**

Yoshitaka Ando ^1, +^, Mirai Yamazaki ^2, +^, Hiroya Yamada ^3,^ *, Eiji Munetsuna ^4^, Ryosuke Fujii ^5^, Genki Mizuno ^6^, Naohiro Ichino ^7^, Keisuke Osakabe ^7^, Keiko Sugimoto ^7^, Hiroaki Ishikawa ^1^, Koji Ohashi ^1^, Ryoji Teradaira^1^, Yoshiji Ohta ^8^, Nobuyuki Hamajima ^9^, Shuji Hashimoto ^3^, Koji Suzuki ^5,^ *.

| miRNAs |  | ρ |  | *P* value |
| --- | --- | --- | --- | --- |
| miR-20a |  | -0.07 |  | 0.13 |
| miR-27a |  | -0.05 |  | 0.33 |
| miR-126 |  | -0.12 |  | 0.01 |

**Supplementary Table S1.** Correlation between FIB-4 score and circulating miRNAs levels.

Date are assessed by Spearman's rank correlation tests.

|  |  | Liver fibrosis grades by FIB-4 score | | | | | | | | |
| --- | --- | --- | --- | --- | --- | --- | --- | --- | --- | --- |
|  |  | Non-fibrosis |  | Possbile fibrosis | | |  | Advanced fibrosis | | |
|  |  | 95% CIs |  | 95% CIs |  | *P* value |  | 95% CIs |  | *P* value |
| miR-20a |  | 1.0 (0.38-3.48) |  | 0.93 (0.30-2.95) |  | 0.31 |  | 1.45 (0.39-3.34) |  | 0.99 |
| miR-27a |  | 1.0 (0.45-4.34) |  | 0.96 (0.38-3.79) |  | 0.25 |  | 1.91 (0.43-4.43) |  | 0.88 |
| miR-126 |  | 1.0 (0.44-2.75) |  | 0.79 (0.34-1.72) |  | 0.03 |  | 1.16 (0.45-1.90) |  | 0.65 |

**Supplementary Table S2.** Association between circulating miRNAs and liver fibrosis grades by FIB-4 score.

Date are expressed as genometric mean value (25th-75th percentiles) and compared by Wilcoxon tests.
